# Trends and Trajectories in the Rise of Large Language Models in Radiology: Scoping Review

**DOI:** 10.2196/78041

**Published:** 2025-12-09

**Authors:** Adhari Al Zaabi, Rashid Alshibli, Abdullah AlAmri, Ibrahim AlRuheili, Syaheerah Lebai Lutfi

**Affiliations:** 1 Human and Clinical Anatomy Department, College of Medicine and Health Sciences, Sultan Qaboos University, P.O. Box 35, Al Khodh, Muscat, 123, Oman; 2 College of Medicine and Health Sciences, Sultan Qaboos University, Muscat, Oman; 3 Medical Education and Informatics Department, College of Medicine and Health Sciences, Sultan Qaboos University, Muscat, Oman

**Keywords:** large language models, GPT-4, scoping review, natural language processing, report generation, clinical decision support, workflow optimization, artificial intelligence, AI, radiology

## Abstract

**Background:**

The use of large language models (LLMs) in radiology is expanding rapidly, offering new possibilities in report generation, decision support, and workflow optimization. However, a comprehensive evaluation of their applications, performance, and limitations across the radiology domain remains limited.

**Objective:**

This review aimed to map current applications of LLMs in radiology, evaluate their performance across key tasks, and identify prevailing limitations and directions for future research.

**Methods:**

A scoping review was conducted in accordance with the framework by Arksey and O’Malley framework and the PRISMA-ScR (Preferred Reporting Items for Systematic Reviews and Meta-Analyses extension for Scoping Reviews) guidelines. Three databases—PubMed, ScopusCOPUS, and IEEE Xplore—were searched for peer-reviewed studies published between January 2022 and December 2024. Eligible studies included empirical evaluations of LLMs applied to radiological data or workflows. Commentaries, reviews, and technical model proposals without evaluation were excluded. Two reviewers independently screened studies and extracted data on study characteristics, LLM type, radiological use case, data modality, and evaluation metrics. A thematic synthesis was used to identify key domains of application. No formal risk-of-bias assessment was performed, but a narrative appraisal of dataset representativeness and study quality was included.

**Results:**

A total of 67 studies were included. (n/N, %)GPT-4 was the most frequently used model (n=28, 42%), with text-based corpora as the primary type of data used (n=43, 64%). Identified use cases fell into three thematic domains: (1) decision support (n=39, 58%), (2) report generation and summarization (n=16, 24%), and (3) workflow optimization (n=12, 18%). While LLMs demonstrated strong performance in structured-text tasks (eg, report simplification with >94% accuracy), diagnostic performance varied widely (16%-86%) and was limited by dataset bias, lack of fine tuning, and minimal clinical validation. Most studies (n=53, 79.1%) had single-center, proof-of-concept designs with limited generalizability.

**Conclusions:**

LLMs show strong potential for augmenting radiological workflows, particularly for structured reporting, summarization, and educational tasks. However, their diagnostic performance remains inconsistent, and current implementations lack robust external validation. Future work should prioritize prospective, multicenter validation of domain-adapted and multimodal models to support safe clinical integration.

## Introduction

The integration of artificial intelligence (AI) into health care has accelerated over the past decade, with large language models (LLMs) emerging as transformative tools for natural language processing in clinical contexts. Built on transformer architectures, models such as GPT-4, bidirectional encoder representations from transformers (BERT), and Text-to-Text Transfer Transformer (T5) have demonstrated high performance in text-based tasks such as summarization, classification, and information extraction across general and clinical domains [1].

Radiology is inherently data intensive and text rich, making it an ideal domain for the application of LLMs. These models can support a wide range of tasks, including automated report generation, structured documentation, code assignment, and even preliminary diagnostic reasoning from clinical narratives [2-5] Despite the growing number of pilot studies, there is no unified synthesis evaluating the practical effectiveness, integration readiness, and safety implications of LLMs in real-world radiology settings.

Several prior scoping reviews have investigated the use of LLMs in radiology, but these have typically focused on specific application domains. For example, Reichenpfader et al [6] performed conducted a scoping review focused exclusively on information extraction from radiology reports. Their analysis highlighted that most approaches relied on encoder-based transformer models such as BERT, that datasets were often small and single center, and that performance varied substantially by annotation quality and task definition. They concluded that, while information extraction is promising, generalizability and external validation are lacking [6,7]. Busch et al [8] conducted a narrative overview of approximately 10 studies specifically addressing structured reporting in radiology. They emphasized the potential of GPT-3.5 and GPT-4 to transform free text into structured templates and discussed opportunities for multilingual structured reporting adoption. Their analysis was conceptual, with limited systematic synthesis across tasks. Nakaura et al [9] traced the evolution of deep learning and transformer architectures in radiology; explained key limitations such as hallucinations, bias, and lack of explainability; and emphasized the risks of premature deployment in clinical decision support. Their review highlighted proof-of-concept applications, including report generation, translation of radiology reports into plain language, exam preparation, and early feasibility of protocol selection and research support [9].

Unlike these prior reviews that were narrowly focused on single use cases (information extraction or patient-facing report simplification), our study systematically mapped the full spectrum of LLM applications across radiology—including decision support, report generation, workflow optimization, and education. Furthermore, our work integrated both generative and nongenerative transformer models, multimodal applications, and educational and operational use cases. This broader lens allowed us to identify converging themes; quantify distribution across modalities; and highlight gaps in validation, equity, and clinical integration. Accordingly, this review aimed to systematically map the applications of LLMs in radiology; evaluate their reported outcomes; and provide a thematic synthesis of emerging use cases, methodological trends, and future research priorities.

## Methods

### Study Design

This scoping review was conducted in accordance with the methodological framework proposed by Arksey and O’Malley [10] and adhered to the PRISMA-ScR (Preferred Reporting Items for Systematic Reviews and Meta-Analyses extension for Scoping Reviews) checklist (Checklist 1) to ensure methodological transparency and reproducibility.

### Eligibility Criteria (PICOS-Based)

Eligibility criteria were defined using the population, intervention, comparator, outcomes, and study designPICOS framework (Table 1). We included peer-reviewed empirical studies evaluating LLM applications in radiology workflows using models such as GPT-3 and GPT-4, BERT, or domain-specific transformers. Reviews, opinion pieces, and conference abstracts were excluded. Only English-language studies published between January 2022 and December 2024 were included due to resource limitations, which we acknowledge may restrict the generalizability of the findings.

**Table 1.** Eligibility criteria for study selection structured using the PICOS framework (population, intervention, comparator, outcomes, study design) with additional filtering criteria related to language and publication date.

PICOS domain or criterion	Inclusion criteria	Exclusion criteria
Population	Studies involving radiology professionals, radiological workflows, or radiology-related data	Studies unrelated to radiology or without reference to radiological applications
Intervention	Use or evaluation of LLMs^a^, including GPT-3 and GPT-4, BERT^b^, or custom transformer models	Studies using general AI^c^ models without a language modeling component
Comparator	—^d^	—
Outcomes	Reported outcomes related to LLM performance, feasibility, integration, or limitations in radiology	Studies lacking outcome data or reporting only theoretical frameworks without application
Study design	Peer-reviewed empirical studies (qualitative, quantitative, or mixed methods)	Reviews, editorials, opinion pieces, letters, and conference abstracts
Language	English	Non-English
Publication date	Published between January 2022 and December 2024	Published before 2022 or after December 2024

a LLM: large language model.

b BERT: bidirectional encoder representations from transformers.

c AI: artificial intelligence.

d Not applicable (scoping review design).

### Information Sources and Search Strategy

The databases were selected to ensure coverage across clinical (PubMed), multidisciplinary (Scopus), and technical and engineering (IEEE Xplore) domains. The search combined MeSH (Medical Subject Headings) and free-text terms related to LLMs (“large language model,” “GPT,” “BERT,” and “transformer-based AI”) and radiology (“radiology,” “medical imaging,” and “diagnostic imaging”).

Database-specific search strings tailored to syntax and operators are provided in Multimedia Appendix 1. Gray literature (eg, arXiv and medRxiv) and conference proceedings were excluded, which may have limited capture of emerging non–peer-reviewed work. Furthermore, the use of MeSH terms in PubMed was optimized but may not have fully captured all relevant variations due to evolving terminology in this rapidly developing field. These limitations may have affected the comprehensiveness of the search and should be considered when interpreting the findings.

### Study Selection

All retrieved records were imported into Rayyan [11] (Qatar Computing Research Institute), a web-based tool designed to facilitate systematic and scoping review workflows. Rayyan facilitated duplicate removal and blinded screening. Two reviewers (AA and IR) independently screened titles and abstracts and assessed full texts against the eligibility criteria. Disagreements were resolved through consensus or, if needed, by a third reviewer (RS). To ensure calibration, an initial pilot screening was conducted, and a random 20% sample of the included studies was cross-checked. The study selection process is presented in the PRISMA (Preferred Reporting Items for Systematic Reviews and Meta-Analyses) 2020 flow diagram.

### Data Extraction Strategy

A structured data extraction form was developed and piloted on a sample of 5 studies. The following data were collected:

- Publication details (year, country, and journal)
- LLM type (eg, GPT-3.5, GPT-4, BERT, or domain-specific models)
- Radiology use case (eg, classification, report generation, decision support)
- Data modalities (text, images, multimodal, or radiology information systems [RISs])
- Evaluation metrics (eg, accuracy, bilingual evaluation understudy [BLEU], recall-oriented understudy for gisting evaluation [ROUGE], Matthews correlation coefficient, area under the curve, and *F*_1_-score)
- Dataset characteristics (size, source, and multicenter vs single center)
- Reported outcomes and limitations

Data extraction was conducted independently by 2 reviewers. A random 20% subset was cross-checked for accuracy, with discrepancies resolved through consensus.

### Secondary Data Extraction and Thematic Classification

#### Data Extraction and Coding

A hybrid thematic analysis was conducted. Initially, themes were extracted manually by 3 independent raters who analyzed and categorized the data. An interrater reliability measure (percentage of agreement) was applied to ensure consistency across raters. Subsequently, GPT-4 was used to assist with clustering recurring patterns using a zero-shot prompt. The prompt applied was as follows: “Act as a pseudo analyst, read this file (Excel file with the raw data), and label abstracts with relevant codes. Provide a summary of recurring themes.”

The outputs generated by GPT-4 were then compared and triangulated with the manually derived results by an additional expert reviewer, who was provided with (1) the original raw Microsoft Excel file and (2) GPT-4’s preliminary coding and theme map. Discrepancies between manual and AI-assisted outputs were discussed in a consensus meeting, and revisions were made to finalize the thematic framework.

It should be noted that GPT-4 was not used during the initial manual theme extraction, which was conducted independently by the 3 student raters. The use of GPT-4 in the subsequent phase was intended to support rather than replace human analytical judgment and ensure that AI-generated outputs were critically appraised before integration.

#### Theme Development

Through inductive synthesis, the extracted codes were grouped into broader categories that reflected the primary ways in which LLMs are currently being explored in radiology. After multiple rounds of refinement, three overarching themes were established: (1) decision support, including diagnostic support, case prioritization, and aiding clinical judgments; (2) report generation, encompassing drafting, summarization, and improving clarity or standardization of radiology reports; and (3) workflow optimization, referring to efficiency gains such as automating routine tasks, assisting communication, and integrating radiology processes into clinical workflows.

This thematic classification was not predetermined but derived from recurring patterns across the reviewed material. GPT-4 was used as a supporting tool to enhance coding efficiency and cross-check clustering of concepts, whereas the final themes were reviewed, validated, and confirmed manually by the research team.

By systematically identifying and categorizing these themes, the analysis provided a structured synthesis of the literature while ensuring methodological transparency and reproducibility.

### Narrative Quality Assessment

Although a formal risk-of-bias assessment was not performed in accordance with scoping review methodology, a narrative appraisal revealed several recurring limitations in the included studies. Many were small-scale, single-institution implementations or proof-of-concept projects, with limited external validation. Most lacked robust methodological descriptions or standardized evaluation metrics, making cross-study comparisons challenging.

In terms of dataset size and representativeness, several studies relied on relatively small or synthetic datasets, often drawn from publicly available repositories rather than real-world clinical systems. This raises concerns about generalizability. Geographically, a substantial proportion of the studies originated from North America, Europe, and China, indicating potential regional bias in the development and evaluation of LLMs for radiology. There was limited representation from low- and middle-income countries, which may affect the global applicability of the findings.

### Critical Reflection on Methodology

This review used a rigorous and transparent methodology; however, certain limitations must be acknowledged. Restriction to English-language studies and the exclusion of gray literature may have limited comprehensiveness. The fast pace of LLM development also means that new studies may have emerged since the search was conducted. Finally, thematic synthesis, while appropriate for mapping breadth, is interpretive and may introduce subjectivity despite the use of calibration and consensus procedures.

## Results

### Overview of the Included Studies

**Figure 1.**
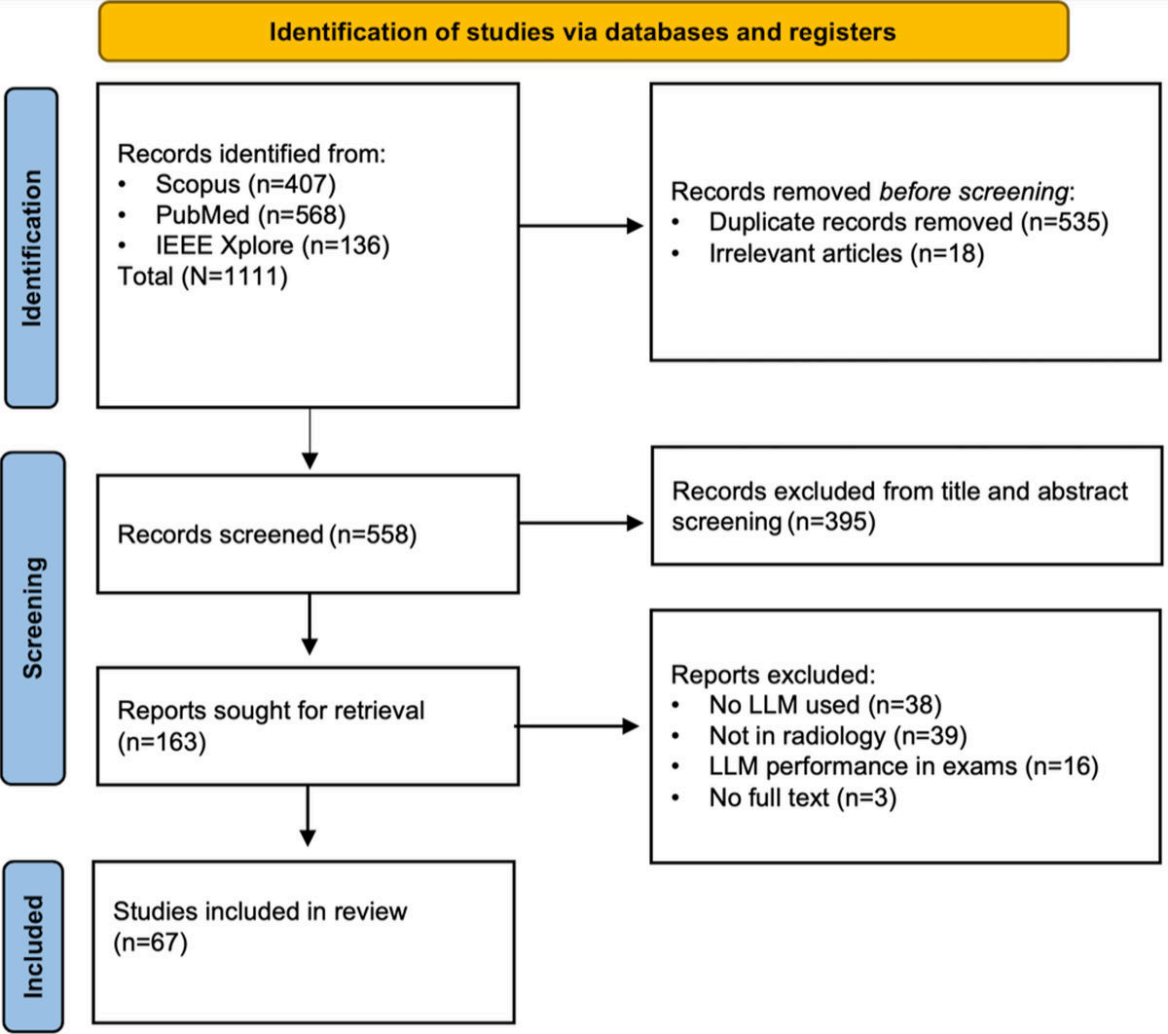
PRISMA (Preferred Reporting Items for Systematic Reviews and Meta-Analyses) 2020 flow diagram detailing the study selection process for the included records across databases. LLM: large language model.

A total of 1111 records were retrieved from Scopus (n=407, 36.6%), PubMed (n=568, 51.1%), and IEEE Xplore (n=136, 12.2%). Of these 1111 records, after removing 535 (48.2%) duplicates and 18 (1.6%) irrelevant records, 558 (50.2%) studies remained. Following title and abstract screening, 163 full-text articles were reviewed, and 67 (41.1%) met the inclusion criteria (Figure 1). A summary of all included articles is presented in Multimedia Appendix 2.

**Figure 2.**
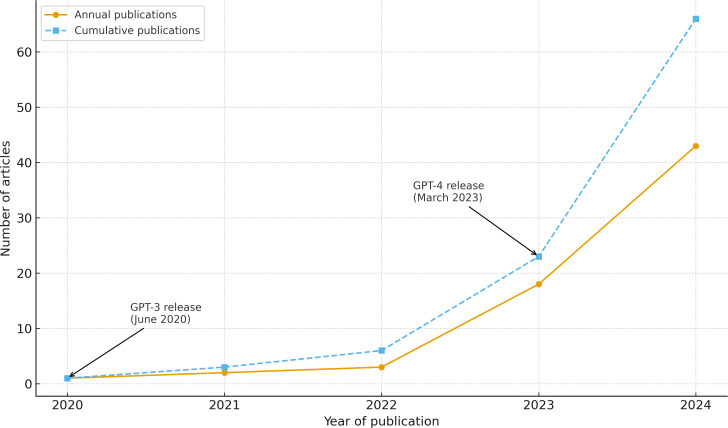
Annual and cumulative number of publications applying large language models in radiology (2020‐2024). Data derived from the included studies (N=67). Milestones for the release of GPT-3 (June 2020) and GPT-4 (March 2023) are annotated.

Most studies (44/67, 65.7%) were published in 2024, reflecting a sharp rise in interest following the release of GPT-4 in March 2023 (Figure 2). Geographically, the United States contributed the most studies (24/67, 35.8%), followed by Japan (10/67, 14.9%) and Germany (10/67, 15%). Very few studies originated from low- and middle-income countries, and a few studies assessed non–English-language corpora (Multimedia Appendix 2).

### Types of LLMs and Implementation Approaches

GPT-4 was the most frequently studied model (28/67, 42%), followed by GPT-3.5 (14/67, 21%). A smaller proportion (n/N, %)used BERT-based models such as CheXbert and BioBERT or domain-specific variants, including Radiology-Llama2 and RadSpaT5. Multimodal models capable of integrating text and images were reported in 17.9% (12/67) of the studies, although few underwent clinical validation.

Regarding input data, 64% (43/67) of the studies used text-based corpora such as radiology reports, request forms, or quizzes; 15% (10/67) analyzed images; 18% (12/67) used multimodal datasets; and 3% (2/67) used either RIS data or exam question datasets (Multimedia Appendix 3). Of the 67 studies, 56 (84%) used English-language corpora (English language only: n=50, 89%; mixed English+another language: n=6, 11%), and 11 (16%) used only corpora in non-English languages (German: n=4, 36%; Japanese: n=4, 36%; Italian: n=2, 18%; French: n=1, 9%). Most studies (53/67, 79%) were single center, whereas 21% (14/67) were multicenter.

### Imaging Modalities and Radiological Subspecialties

**Figure 3.**
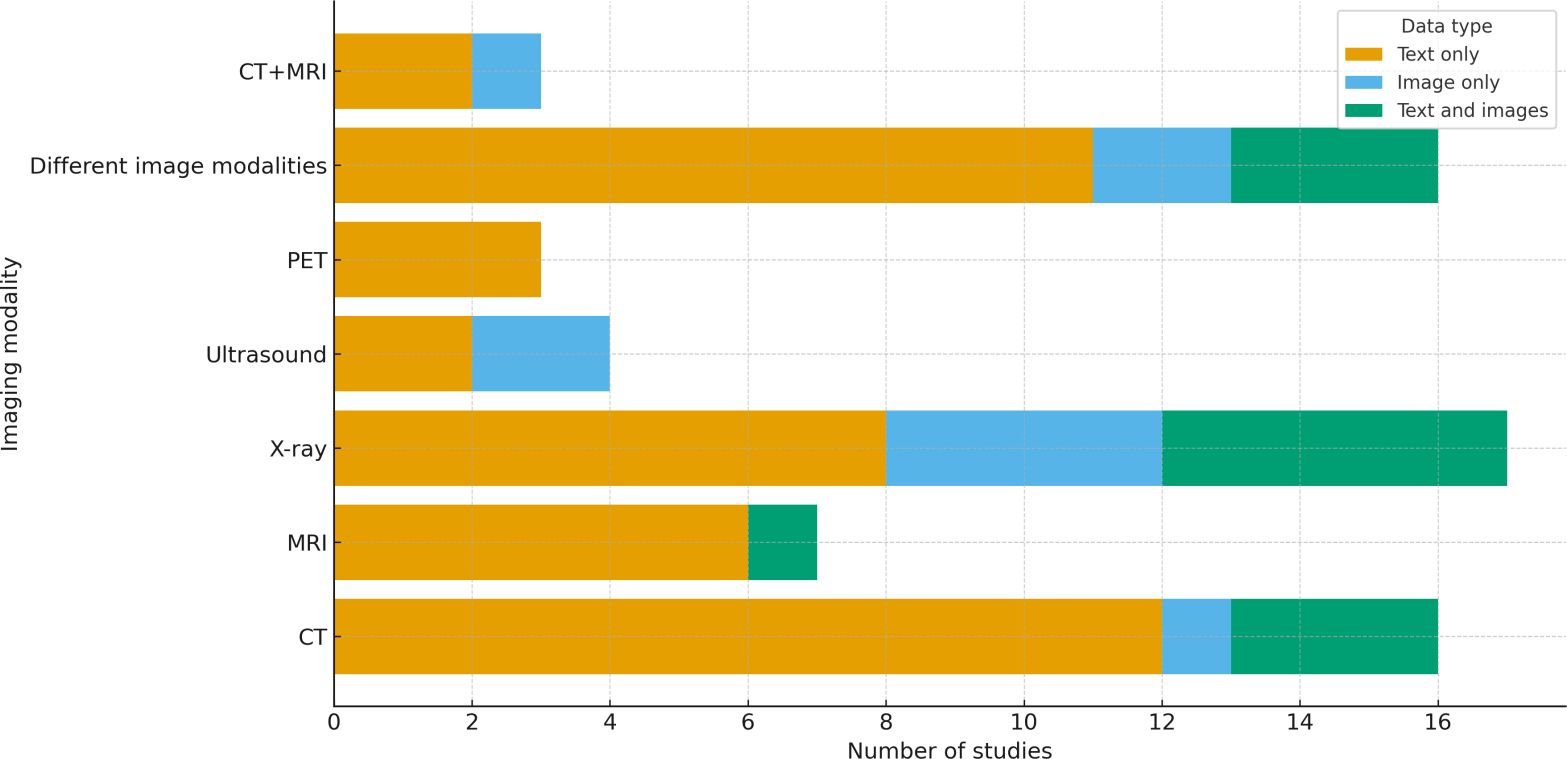
Imaging modalities used, stratified by data type (N=67). Most studies relied on text-only data (yellow), with fewer using image-only (blue) or multimodal text+image datasets (green) datasets. CT: computed tomography; MRI: magnetic resonance imaging; PET: positron emission tomography.

Imaging modality use varied across the studies (Figures3  4). Multimedia Appendix 4 shows the distribution of the 67 studies across various radiology subspecialties. The most represented field was thoracic imaging with 24% (16/67) of the studies, followed by general radiology (13/67, 19%) and oncologic imaging (11/67, 16%).

**Figure 4.**
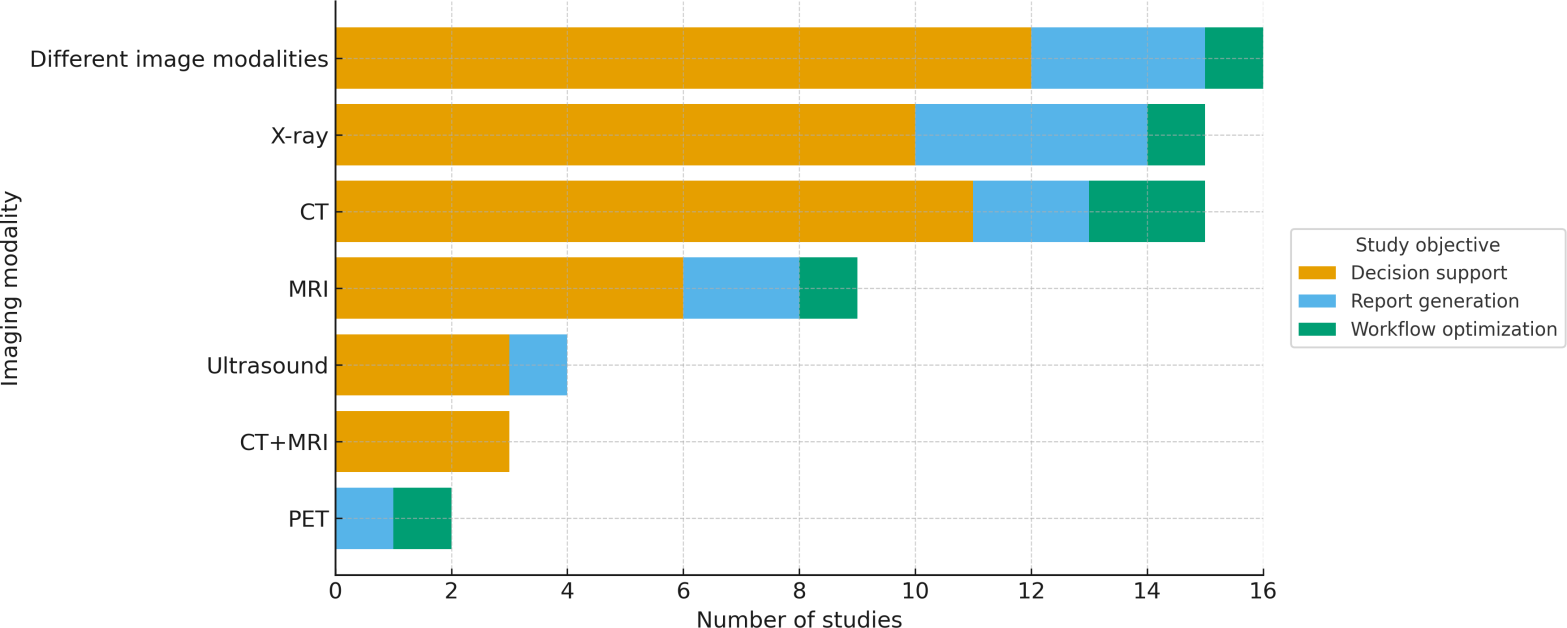
Imaging modality by study objective (N=67). Decision support (yellow) predominated, followed by report generation (blue) and workflow optimization (green). Positron emission tomography (PET) and ultrasound were the least represented. CT: computed tomography; MRI: magnetic resonance imaging.

### Thematic Domains of Application

#### Overview

Table 2 shows the 3 thematic domains that emerged (detailed thematic domains and models are presented in Multimedia Appendix 5).

**Table 2.** Thematic classification of large language model applications in radiology across the 67 included studies (2022‐2024).

Theme and subtheme	Articles
Decision support	
Classification	Blankemeier et al [12] Chambon et al [13] Fervers et al [14] Haver et al [15] Olivato et al [16] Putelli et al [17] Santos et al [18] Sehanobish et al [19] Suzuki et al [20] Wu et al [21] Zhang et al [22]
Diagnosis from clinical cases	Danu et al [23] Horiuchi et al [24] Horiuchi et al [25] Kurokawa et al [26] Wada et al [27]
Diagnosis from images	Elek et al [28] Khare et al [29] Pachade et al [30] Busch et al [31] Silva et al [32] Wu et al [33] Kottlors et al [34] Overgaard Olesen et al [35] Lee et al [36] Reith et al [37] Horiuchi et al [38]
Extracting information from reports	Mukherjee et al [39] Bressem et al [4] Tan et al [40] Tay et al [41] Russe et al [42] Le Guellec et al [43] Lybarger et al [44] Dada et al [45] Sun et al [46] Bhayana et al [47]
Summarization	Wu and Bibault [48]
Report generation	
Generating the report	Danu et al [49] Hasani et al [50] Ji et al [51] López-Úbeda et al [52] Mallio et al [53] Moezzi et al [54] Nakaura et al [55] Selivanov et al [56] Shentu and Al Moubayed [57] Soleimani et al [58] Woźnicki et al [59] Wu et al [60] Bhayana et al [61] Tie et al [62]
Summarization	Karn et al [63]
Quality of complex reports	Zhu et al [64]
Workflow optimization	
Selecting appropriate modality from radiology order	Gertz et al [65]
Image quality	Chen et al [66]
Exam questions	Mistry et al [67]
Summarization	Nishio et al [68]
Classification	Yasaka et al [69] Huemann et al [70] Kanzawa et al [71]
User interface improvement	Zhang et al [72]
Identification of reports containing recommendations	Abbasi et al [73]
Detection of errors	Kathait et al [74]
Simplification of reports for patients	Sarangi et al [75]
Answering patient questions	Rogasch et al [76]

#### Theme 1: AI-Assisted Clinical Decision Support

Four subthemes emerged from this theme.

##### Classification Tasks

Across radiology classification tasks, domain-tuned transformers remained the most reliable, whereas general LLMs were mixed. BERT-style models standardized the Thyroid Imaging Reporting and Data System and matched or exceeded radiologists for chest x-ray report extraction [18,22], with added interpretability and effectiveness in Italian reports [17]. GPT-3.5 and GPT-4 underperformed or were inconsistent for the Liver Imaging Reporting and Data System and tumor node metastasis staging [14,20], although structured Reporting and Data System categorization showed promise [21]. The multimodal GPT-4V struggled to describe Breast Imaging Reporting and Data System features [15], whereas specialized models such as RadBERT and a 3D vision language model (Merlin) achieved strong document-level COVID-19 classification and surpassed other models. Overall, BERT-family and domain-adapted approaches are currently more dependable than generic LLMs for clinical deployment.

##### Diagnosis From Clinical Cases

Across clinical case diagnosis, general LLMs remained inconsistent and typically trailed expert radiologists. GPT-4 reached approximately 50% overall accuracy on neuroradiology cases of the week, performing far worse on central nervous system tumors (16%) than on non–central nervous system tumors (62%) [23]. In musculoskeletal cases, text-only GPT-4 was roughly at the resident level but below board-certified radiologists, whereas GPT-4V lagged further [23]. On challenging cases from the Freiburg Neuropathology Case Conference, both GPT-4 and GPT-4V underperformed compared to radiologists [24]. Among Anthropic models, Claude 3.5 Sonnet outperformed Claude 3 Opus, with accuracy improving when both clinical history and imaging were provided, yet differential diagnosis listing remained limited [26]. Targeted prompt engineering and confidence thresholds measurably boosted GPT-4 Turbo’s diagnostic accuracy, highlighting the value of workflow tuning [27]

##### Diagnosis From Images

General LLMs were promising but not yet dependable. GPT-4 (via Bing) was able to recognize basic computed tomography (CT) and magnetic resonance imaging (MRI) features but lacked diagnostic reliability [28]. Multimodal and domain-tuned models fared better: mmBERT set a new visual question answering state of the art with interpretable attention maps [29], and self-supervised Contrastive Language-Image Pretraining improved large-vessel occlusion detection over supervised baselines [30]. GPT-4V showed potential across subspecialties but should complement clinicians, not replace them [31], and GPT-3.5 showed variable accuracy and should be considered as supplementary—not stand-alone—for dental panoramic radiographs [32]. Pairing LLMs with image-to-text modules boosted diagnostic performance in thyroid ultrasound [33]. For differential diagnosis, GPT-4 reached 68.8% concordance with experts (93.8% of outputs were acceptable), with best results in neuroradiology and chest x-rays, yet task performance varied [34] and remained limited for specific findings such as pulmonary congestion [35]. Broadly, LLMs were able to propose differentials but were not reliable for independent use [38]; specialized vision models such as KARA-CXR currently outperform ChatGPT in chest x-ray interpretation [36]. GPT-4, even with single-shot prompts, identified incidental findings with high precision and recall from CT scans. In contrast, multimodal LLMs remain inadequate for pediatric image interpretation [37].

##### Extracting Information From Reports

Domain-tuned transformers consistently excelled. BERT variants, especially RadBERT, surpassed other text report classifiers with less annotation in extracting findings from intensive care chest radiograph reports [4], and SpERT achieved high anatomy-linked extraction [44]. Large clinical models also performed strongly: GatorTron reached high accuracy for cancer disease response [40], and an information extraction pipeline inferred metastatic sites accurately and explainably [41]. The open-source Vicuna showed excellent accuracy on emergency brain MRI reports without additional training [43].

### Theme 2: LLMs for Report Generation and Quality

In total, 22.4% (15/67) of the studies examined LLMs for generating, structuring, or evaluating radiology reports, falling into 2 streams.

#### Text to Text

These systems converted free text into structured outputs or summaries: T5 and SciFive performed relation extraction to produce clinician-interpretable structured reports [54], fine-tuned T5 yielded near-expert MRI conclusions in Spanish [52], and Llama 2-70B locally structured reports with approximate human accuracy but variable semantics across languages and findings [59]. GPT-4 improved standardization and generated reports with higher clarity and conciseness than those of human reports but lower diagnostic precision [50,55,58]. It produced the most reliable report templates versus Perplexity, GPT-3.5, and Bing [53]. PEGASUS generated clinically acceptable personalized positron emission tomography (PET) impressions [62].

#### Image to Text

These pipelines enhanced captioning and paired reports. CXR-IRGen outperformed baselines for chest x-ray image-report pairs [54,57], and a Bloomz-7B1 2-step model (image→abnormality→report) was promising and has potential to reduce workload [49]. GPT-4 consistently emerged as the most robust model across multiple benchmarks [55,58], offering both high readability and coherence, although challenges in diagnostic precision and handling rare findings remain. All 4 studies in this theme showed that LLMs matched or exceeded baseline performance metrics such as BLEU, ROUGE, and Consensus-Based Image Description Evaluation for radiology report generation [49,51,56,57]. Integration of domain-adaptive training or prompt tuning improved model performance, underscoring the importance of radiology-specific fine-tuning.

### Theme 3: Clinical Workflow Optimization

total of 17.9% (12/67) of the studies evaluated how LLMs can optimize various nondiagnostic tasks in clinical workflows. This theme included 6 subthemes.

#### Summarization and Simplification

LLMs supported patient‐facing and clinician‐to‐clinician communication. GPT-3.5 reliably simplified radiology reports into plain language while preserving salient clinical details [75]. Text-to-text transformers (eg, RadSpaT5 and T5) achieved expert-level abstractive summaries, producing accurate report conclusions in most cases [68].

#### Classification of Reports

Fine-tuned BERT models accurately categorized brain MRI reports into treatment-related groups and identified lung cancer pretreatment cases with performance comparable to that of human experts [69,71]. Domain-adapted variants (BioClinicalBERT and RadBERT) further improved PET and CT report classification, highlighting the value of specialty-specific pretraining [70].

#### Error Detection and Recommendation Extraction

LLMs showed high precision in identifying diagnostic inaccuracies and extracting actionable recommendations. The Augmented Transformer Assisted Radiology Intelligence model, which integrates both vision and language processing, significantly outperformed traditional natural language processing approaches in detecting laterality errors within reports [74]. A BERT-based model identified reports containing recommendations for additional imaging with high precision and recall, enabling automated recommendation extraction [73].

#### Radiology Protocol Selection and Answering Patient Queries

GPT-4 accurately selected imaging modalities and protocols from referral forms, indicating potential to streamline protocoling tasks [65]. It also answered common patient questions regarding PET and CT preparation and reporting as a supplementary education tool [76].

#### User Interface Enhancement

User interface enhancement was explored through models such as ChatUI-RIS, which improved the usability of RISs by offering a more intuitive interface and enhanced learning experiences, particularly for trainees and junior radiologists [72].

#### Image Quality Assessment and Educational Use

Multimodal LLMs with visual understanding (eg, IQAGPT) provided effective CT image quality assessment [66]. For education, GPT-4 generated high-quality board-style multiple-choice questions (ie, questions at the level of those on a board examination) and rationales for radiology curricula [67].

### Model Performance Across Applications

Performance varied widely across tasks (Table 3; the full metrics can be found in MultimediaAppendices 2  6). Models fine-tuned on domain-specific corpora (eg, RadBERT, BioClinicalBERT, and Japanese BERT variants) consistently outperformed general-purpose LLMs in structured classification and report-based tasks, often achieving accuracies of >95% [69,71,73].

**Table 3.** Summary of performance ranges across the included studies. The lowest and highest reported values are shown where available. Data were extracted from MultimediaAppendices 2^4^ (N=67).

Task or application domain and metric	Reported range
Classification	
Accuracy (%)	83‐97
*F*_1_-score	0.66‐1.00
AUC^a^	0.84‐0.99
Diagnostic reasoning from clinical cases	
Accuracy (%)	16‐50
Diagnosis from images	
Accuracy (%)	25‐84
Match rate (%)	48-62
Concordance (%)	66.7-68.8
Information extraction from radiology reports	
Accuracy (%)	83-97
*F*_1_-score	0.66‐1.00
AUC	0.84-0.99
Report generation and summarization	
*F*_1_-score	0.29-0.88
Accuracy (%)	67-89
Clinical acceptability (physician rated; %)	89
BLEU^b^ or ROUGE^c^ scores	Variable, generally modest (BLEU: 0.46‐0.74; ROUGE-L^d^: 0.37‐0.61)
Similarity score (%)	98.9-99.3
Quality assessment	
Accuracy (%)	70.2-98.3

a AUC: area under the curve.

b BLEU: bilingual evaluation understudy.

c ROUGE: recall-oriented understudy for gisting evaluation.

d ROUGE-L: recall-oriented understudy for gisting evaluation based on the longest common subsequence.

In contrast, performance for diagnostic reasoning and image-based tasks remained modest. For instance, GPT-4V achieved only 27% to 35% accuracy in primary and differential diagnoses [31], and GPT-4 variants reached <25% accuracy in case-based diagnostic challenges [23].

Text-based applications such as error detection [74] and structured report inference [18,73] approached human-level accuracy (≥95%). Image-focused tasks yielded lower values, with rank-1 accuracy as low as 25% [32], area under the curve values between 0.80 and 0.83 [30,33], and *F*_1_-scores below 0.30 in some generative settings [57].

Report generation and simplification tasks demonstrated variable performance depending on evaluation metrics. While BLEU and ROUGE scores remained modest, physician-rated acceptability and utility scores were encouraging [62,77], suggesting that automated metrics may underestimate clinical usability. GPT-4 also showed superior performance in exam question generation [67] and summarization [75].

## Discussion

### Principal Findings

#### Overview

This scoping review provides the first comprehensive synthesis of LLM applications across all domains of radiology. By mapping 67 studies, we identified 3 main areas of application: clinical decision support, report generation, and workflow optimization. There is evidence suggesting that LLMs are most reliable in structured tasks such as classification, information extraction, and educational support, whereas diagnostic reasoning and visual interpretation remain underdeveloped.

#### Decision Support

GPT-based and BERT models showed strong performance in structured classification tasks such as the Thyroid Imaging Reporting and Data System, the Liver Imaging Reporting and Data System [14,15,18,21], fracture coding [42], and tumor node metastasis staging [20], particularly when domain-specific BERT variants were fine-tuned on radiology data. These models frequently matched or exceeded human performance in multilingual and specialty-specific contexts. In contrast, diagnostic reasoning tasks involving clinical cases or direct image interpretation showed limited and inconsistent performance. General-purpose GPT-4 and GPT-4V models achieved variable accuracy across case-based and imaging tasks, underscoring the immaturity of current multimodal reasoning [15,24,25,27,31].

#### Report Generation

Transformer models such as T5, PEGASUS, and GPT-4 generated radiology reports that were linguistically coherent and frequently rated as clinically acceptable. Physician-rated outcomes often aligned GPT-4 reports with radiologist-written impressions. However, hallucinations and factual inaccuracies persist, particularly in rare or ambiguous cases. Automated linguistic metrics (BLEU and ROUGE) did not always correlate with clinical usability, highlighting the importance of human-centered evaluation. Without factuality scoring and domain-specific safeguards, unsupervised deployment of report generation tools remains premature.

#### Workflow Optimization

While our thematic synthesis identified distinct application domains, we acknowledge that the “workflow optimization” category is intentionally broad. It encompasses a range of nondiagnostic use cases, including patient education, radiology report simplification, imaging protocol selection, and user interface enhancement. This thematic grouping reflects the expanding role of LLMs in supporting communication, training, and clinical efficiency beyond core diagnostic tasks. Although its breadth may resemble a “catch-all,” we believe that it accurately represents the dynamic and evolving integration of LLMs into radiological practice. Notably, the most reliable use cases for near-term clinical integration were concentrated in workflow support tasks. These included report simplification, protocol selection [73], error identification [74], and RIS user interface enhancement [72]. Such tasks rely primarily on structured reasoning and language fluency rather than on complex diagnostic inference, making them especially suitable for early implementation. Specialized tools such as Augmented Transformer Assisted Radiology Intelligence (for error detection) [74] and ChatUI-RIS (for user interface enhancement) [72] outperformed general-purpose LLMs, reinforcing the value of domain adaptation. Educational uses such as generating board-style multiple-choice questions also proved effective, with high user satisfaction and accuracy [67]. Taken together, these low-risk, high-utility functions offer a promising entry point for safe and meaningful adoption of LLMs in radiology.

#### Emerging Trends

Two developments were particularly noteworthy. First, multimodal LLMs integrating text and image inputs are moving toward context-aware systems but continue to show high variability in performance and lack prospective validation. Second, domain-specific models such as Radiology-Llama2 and RadSpaT5 demonstrate stronger alignment with radiological terminology but remain underrepresented. Broader external validation and adoption of these models could improve interpretability and clinical fidelity.

### Methodological Limitations of the Evidence

Several methodological gaps were consistently observed across the literature. Most studies relied on retrospective, single-center datasets, frequently limited to chest radiographs or neuroradiology, restricting generalizability. Sample sizes were often small, and only 22% of the studies (15/67) reported external validation. Publication bias is likely as studies with positive results may be preferentially published. Heterogeneous reporting of metrics further complicates benchmarking, and the absence of standardized evaluation frameworks for radiology-specific tasks prevents direct comparison across studies.

### Equity and Global Applicability

The predominance of English-language publications and Western data sources poses a significant barrier to equitable implementation. Without multilingual evaluation datasets and cross-regional external validation, performance estimates risk being skewed toward English-language and high-resource settings. Ensuring equity and inclusivity in model development and validation is essential for global relevance.

### Recommendations and Future Work

Future research should prioritize the following areas:

1. Data and validation; assemble diverse, multicenter, and multilingual datasets to improve generalizability. Conduct prospective evaluations across clinical environments.
2. Evaluation standards; develop radiology-specific factuality and safety benchmarks and ensure standardized reporting of performance and bias assessments.
3. Human factors; implement human-in-the-loop frameworks for oversight, error mitigation, and usability evaluation.
4. Governance; establish clear regulatory guidance and accountability standards to ensure transparency and safety in clinical use.

### Limitations

This scoping review has several limitations that should be acknowledged to aid interpretation and guide future research.

First, the search strategy, while designed to be comprehensive, was limited to 3 databases: PubMed, Scopus, and IEEE Xplore. These were selected to capture clinical, biomedical, and technical literature; however, this may have excluded relevant studies indexed in other databases (eg, Embase or Web of Science) or reported in gray literature sources such as arXiv and medRxiv or key conference proceedings (eg, NeurIPS and Medical Image Computing and Computer-Assisted Intervention). This limitation may have led to the omission of emerging or unpublished work.

Second, although efforts were made to use both free-text and controlled vocabulary (eg, MeSH terms in PubMed), the evolving and inconsistent terminology used to describe LLMs may have affected search sensitivity. Terms such as “GPT,” “LLM,” or “transformer-based AI” may not have been uniformly used across all relevant publications. While the search was iteratively refined and detailed strategies are included in Multimedia Appendix 1 to improve reproducibility, some studies may have been inadvertently missed due to terminology mismatch.

Third, only English-language articles were included. This decision was made to ensure consistency in interpretation and quality appraisal; however, it introduces language bias and may have excluded valuable contributions from non–English-speaking regions, particularly in a globally active research field such as AI.

Fourth, consistent with the framework by Arksey and O’Malley [10], we did not include a formal quality assessment of the included studies. While appropriate for scoping reviews, future systematic reviews could integrate AI-specific appraisal tools (eg, the Minimum Information About Clinical Artificial Intelligence Modeling checklist and Checklist for Artificial Intelligence in Medical Imaging) to enhance interpretability. Importantly, the performance ranges reported across the studies (Table 3) should be approached with caution due to the heterogeneity of study designs, evaluation metrics, datasets, and model versions. Many included studies had proof-of-concept or single-institution designs with limited generalizability. Without standardized benchmarks or head-to-head comparisons, the reported values are best interpreted as illustrative of the field’s current status rather than definitive benchmarks.

Publication bias is a potential concern, particularly given the rapid growth and high visibility of LLM research. Studies with positive or novel findings may be more likely to be published and indexed, whereas negative or inconclusive results may be underrepresented. Although publication bias was not formally assessed, this limitation should be considered when interpreting the results.

Fifth, while thematic synthesis is useful for structuring a heterogeneous literature, it is inherently interpretive. We mitigated bias by having 2 reviewers code independently and resolve discrepancies through consensus; however, subjective judgment may still have influenced the final thematic map. In addition, studies that addressed multiple tasks were assigned to a single primary category to avoid duplication. Certain subthemes—such as classification—appear under 2 overarching themes (decision support and workflow optimization). This placement reflects differences in the primary intent (eg, classifying reports and images to support diagnosis vs to streamline workflow), as detailed in the Results section. Finally, while the initial thematic analysis was conducted manually by human researchers, GPT-4 was later used as a supportive tool to assist in clustering and cross-verifying patterns. Given that GPT-4 is a generative and nondeterministic model, the reproducibility of its suggested outputs cannot be fully guaranteed. Therefore, this hybrid approach may introduce potential bias and variability, which should be considered when interpreting the thematic synthesis.

### Conclusions

The integration of LLMs into radiology is accelerating but remains uneven across application domains. Structured tasks such as classification and information extraction are approaching maturity, whereas diagnostic reasoning and multimodal interpretation require substantial improvement. Safe clinical deployment will depend not only on technical performance but also on rigorous validation, global inclusivity, and ethical governance.

## Supplementary material

10.2196/78041 Multimedia Appendix 1 Full search strategies.

10.2196/78041 Multimedia Appendix 2 Summary of all the included articles.

10.2196/78041 Multimedia Appendix 3 Data modalities used across the included studies (N=67): text-only (eg, radiology reports, cases, and request forms), image-only (eg, x-ray, computed tomography, and magnetic resonance imaging), multimodal (text + images), and system or metadata sources (eg, radiology information system) sources.

10.2196/78041 Multimedia Appendix 4 Distribution of radiology studies by subspecialty (N=67). This chart illustrates the number of studies conducted in each radiology subspecialty. Thoracic imaging, general radiology, and oncologic imaging were the most frequently studied areas.

10.2196/78041 Multimedia Appendix 5 Summary of the extracted themes from the included articles (N=67).

10.2196/78041 Multimedia Appendix 6 Reported performance metrics of large language model (LLM) applications in radiology across the included studies (N=67).

10.2196/78041 Checklist 1 PRISMA-ScR checklist.

## Abbreviations

AI: artificial intelligence
BERT: bidirectional encoder representations from transformers
BLEU: bilingual evaluation understudy
CT: computed tomography
LLM: large language model
MeSH: Medical Subject Headings
MRI: magnetic resonance imaging
PET: positron emission tomography
PRISMA: Preferred Reporting Items for Systematic Reviews and Meta-Analyses
PRISMA-ScR: Preferred Reporting Items for Systematic Reviews and Meta-Analyses extension for Scoping Reviews
RIS: radiology information system
ROUGE: recall-oriented understudy for gisting evaluation
T5: Text-to-Text Transfer Transformer
