# Supplementary material for: Trends and Trajectories in the Rise of Large Language Models in Radiology: Scoping Review
Source: JMIR Med Inform. 2025 Dec 9;13:e78041. doi: 10.2196/78041 (PMC12688054; doi:10.2196/78041)
Supplement: Multimedia Appendix 1 [file medinform-v13-e78041-s001.docx]

Appendix X. Full Search Strategies

1. PubMed (MEDLINE) Search Strategy

Searched: June 2024

("Large Language Model"[Title/Abstract] OR "LLM"[Title/Abstract] OR "GPT"[Title/Abstract] OR "Generative Pre-trained Transformer"[Title/Abstract] OR "Transformer-based AI"[Title/Abstract])

AND

("Radiology"[MeSH Terms] OR "Radiology"[Title/Abstract] OR "Medical Imaging"[Title/Abstract] OR "Diagnostic Imaging"[MeSH Terms] OR "Diagnostic Imaging"[Title/Abstract])

AND

("2022/01/01"[Date - Publication] : "2024/06/30"[Date - Publication])

2. SCOPUS Search Strategy

Searched: June 2024

(Note: SCOPUS does not support MeSH; search uses TITLE-ABS-KEY for keyword fields)

(TITLE-ABS-KEY("Large Language Model" OR "LLM" OR "GPT" OR "Generative Pre-trained Transformer" OR "Transformer-based AI"))

AND

(TITLE-ABS-KEY("Radiology" OR "Medical Imaging" OR "Diagnostic Imaging"))

AND

(PUBYEAR > 2021 AND PUBYEAR < 2025)

3. IEEE Xplore Search Strategy

Searched: June 2024

("Large Language Model" OR "LLM" OR "GPT" OR "Generative Pre-trained Transformer" OR "Transformer-based AI")

AND

("Radiology" OR "Medical Imaging" OR "Diagnostic Imaging")

Refined by:

- Content Type: Journals, Conferences

- Publication Date: 2022 – 2024

Note to Reviewers:

Search queries were adapted to the syntax and indexing capabilities of each database. In PubMed, MeSH terms were used where available. In SCOPUS and IEEE Xplore, controlled vocabulary was not applicable; keyword searches were used instead. This may have impacted sensitivity and coverage across databases. The search strategy was designed to balance comprehensiveness with specificity and is reported here to allow replication or extension in future updates.
