# Supplementary figures and images for: Trends and Trajectories in the Rise of Large Language Models in Radiology: Scoping Review
Source: JMIR Med Inform. 2025 Dec 9;13:e78041. doi: 10.2196/78041 (PMC12688054; doi:10.2196/78041)

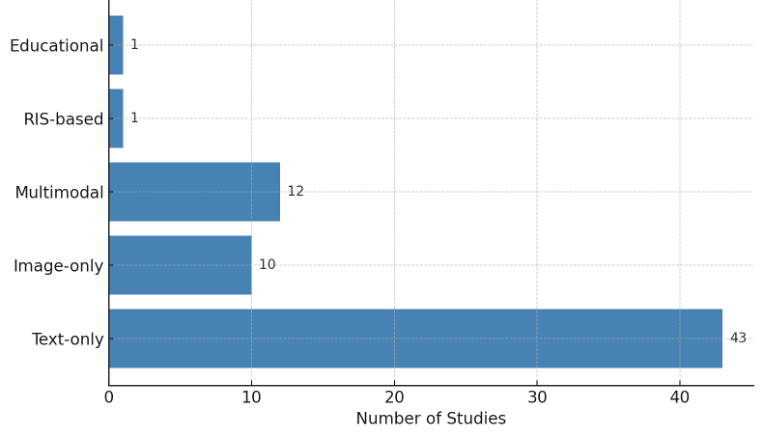

Supplement: Multimedia Appendix 3 [file medinform-v13-e78041-s003.png]

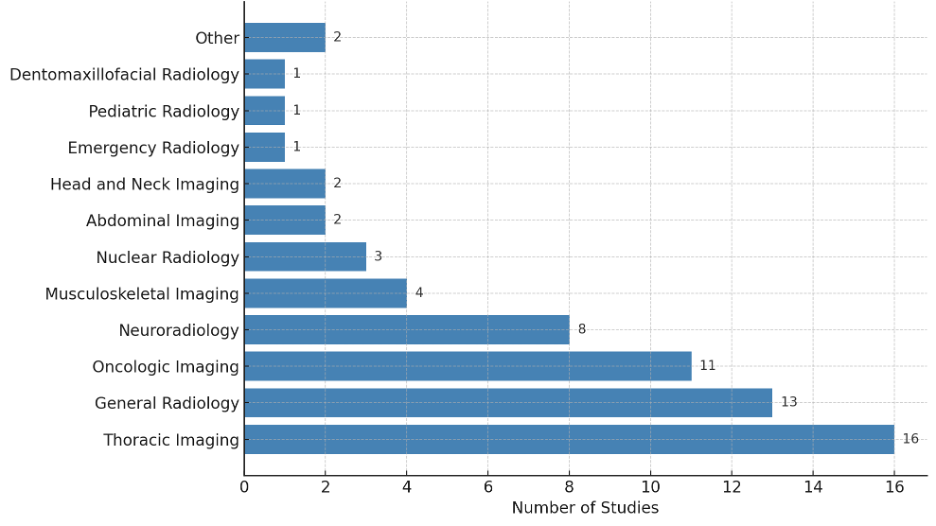

Supplement: Multimedia Appendix 4 [file medinform-v13-e78041-s004.png]
