# Supplementary material for: Trends and Trajectories in the Rise of Large Language Models in Radiology: Scoping Review
Source: JMIR Med Inform. 2025 Dec 9;13:e78041. doi: 10.2196/78041 (PMC12688054; doi:10.2196/78041)
Supplement: Multimedia Appendix 6 [file medinform-v13-e78041-s006.docx]

Table 3: **Reported performance metrics of large language model (LLM) applications in radiology across included studies (n=67).**The table summarizes study-level performance values for accuracy, AUC, and F1 scores, stratified by application domain. Metrics are reported as provided in the original studies, highlighting the heterogeneity of evaluation approaches.

| **Article** | **Model Used** | **Application of LLM** | **Data Type** | **Accuracy (highest reported value)** |
| --- | --- | --- | --- | --- |
| [57] | IQAGPT, ChatGPT, BLIP-2, GPT-4 | Assess image quality | images | 70.2 |
| [49] | Fine-tuned BERT (Japanese model) | classifications of reports | Text | 97 |
| [51] | BioClinicalBERT, RadBERT | Classifications of reports | Text | RadBERT: 77.4 |
| [50] | Fine-tuned BERT (Japanese) | Classifications of reports | Text | 98.3 |
| [61] | GPT-4, BERT, LSTM with Attention | classifications of reports | Text | 92.3 |
| [62] | BERT, ClinicalBERT, PubMedBERT | classifications of reports | Text | PubMedBERT: 92.9 |
| [13] | BERT | classifications of reports | text | 96.1 |
| [58] | GPT-4, Llama 2 | Construct exam questions | Text | LLAMA: 69% , GPT-4: 100% |
| [18] | GPT-4, GPT-4V | Diagnosis from cases | Text | GPT-4 accuracy: 22%, GPT-4V: 16% |
| [27] | ChatGPT-3.5, ChatGPT-4, Bard | Diagnosis from image | Images | 84 |
| [68] | GPT-4, GPT-4V | Diagnosis from reports | Text | GPT-4-based: 43 |
|  | GPT-4V | Diagnosis from reports | Images | GPT-4V: 27% for primary diagnosis, 35% for Diffrential diagnosis |
| [25] |  |  |  |  |
| [53] | BERT | Extract information from reports | Text | 99 |
| [12] | BERT, CNN | Extract information from reports | Text and images | 95.8 |
| [36] | Vicuna (open-source LLM) | Extract information from reports | Text | >95 |
| [73] | Five AI-LLMs (Dolly-v2, Vicuna-13b, Llama-2.0-13b, GPT-3.5, and GPT-4.0) | Extract information from reports | Text and images | 87% (Dolly), 83% (Vicuna), 93% (Llama), 91% (GPT-3.5), 97% (GPT-4.0) |
| [52] | Augmented Transformer Assisted Radiology Intelligence (ATARI) | identifying laterality errors in radiology reports | Text and images | 98.3 |
| [32] | GPT-4, Gemini 1.5 Pro, Claude 3 Opus | Image analysis and interpretation | text and Images | 27.8 |
| [26] | ChatGPT-3.5 | Image analysis and interpretation | Images | Rank-1: 25% Rank-2: 57.14%, and Rank-3: 67.85% |
| [11] | BERT-based fusion NLP model | Inference from report | Text | 98 |
| [14] | GPT-3.5 | Inference from report | Text | 53% from unstructered and 44% from structured data |
| [16] | GPT-3.5, GPT-4, Claude-2 | Inference from report | Text | 75 |
| [34] | GatorTron, BERT, CNN | Inference from report | Text | 89.76 |
| [15] | GPT-4 | Inference from report | text | 73 for T, 91 for N, and 93 for M |
| [17] | GPT-4V | Report generation | Images | 29 |
| [47] | ChatGPT-3.5 | Report simplification/ summarization | Text | 94.7 |
|  |  |  |  |  |
| **Article** | **Model Used** | **Application of LLM** | **Data Type** | **AUC** |
| [24] | CLIP, BERT | Diagnosis from reports | Text and images | 0.8 |
| [27] | ChatGPT-3.5, ChatGPT-4, Bard | Diagnosis from image | Images | 0.83 |
| [61] | GPT-4, BERT, LSTM with Attention | classifications of reports | Text | 0.96 |
| [50] | Fine-tuned BERT (Japanese) | Classifications of reports | Text | 0.97 |
| [49] | Fine-tuned BERT (Japanese model) | classifications of reports | Text | 0.994 |
| [4] | GER-BERT, MULTI-BERT, FS-BERT, RAD-BERT | Extract information from reports | Text | 0.98 for congestion, 0.97 for effusion, 0.97 for consolida- |
|  |  |  |  | tion and 0.99 for pneumothorax, |
| [59] | 3D Vision-Language Model | Image analysis and interpretation | Text and 3D images | .812 |
| [70] | Vicuna-13B | Extract information from reports | Text | 0.84. |
| [12] | BERT, CNN | Extract information from reports | Text and images | Symptomatic patients (0.866), Screened patients (0.891), Clinic patients (0.796). |
|  |  |  |  |  |
| **Article** | **Model Used** | **Application of LLM** | **Data Type** | **F1 score** |
| [43] | CXR-IRGen (combining Vision Module and Language Module with LLM) | Report generation | Text and images | 0.29 |
|  | SpERT (Span-based BERT) | Extract information from reports | Text | 0.66 |
| [12] | BERT, CNN | Extract information from reports | Text and images | 0.703 |
| [72] | German BERT models | extract information from reports | text and images | 0.8397 |
| [40] | GPT-4 | Report generation | Text | 0.8775 |
| [60] | RadBERT | classifications of reports | text | 0.889 |
| [61] | GPT-4, BERT, LSTM with Attention | classifications of reports | Text | 0.893 |
| ([13] | BERT | classifications of reports | text | 0.959 |
| [11] | BERT-based fusion NLP model | Inference from report | Text | 0.99 |
| [53] | BERT | Extract information from reports | Text | 95.2 |
| [59] | 3D Vision-Language Model | Image analysis and interpretation | Text and 3D images | .767 |
| [69] | GPT-3.5 and GPT-4 | Assess report quality | text | 0.4881 |
| [39] | Llama-2-70B-chat | Report generation | Text | 0.7 for English reports, 0.68 for Germen reports |
| [73] | Five AI-LLMs (Dolly-v2, Vicuna-13b, Llama-2.0-13b, GPT-3.5, and GPT-4.0) | Extract information from reports | Text and images | 0.87 (Dolly), 0.84 (Vicuna), 0.86 (Llama), 0.88 (GPT-3.5), 0.88 (GPT-4.0) |
| [74] | GPT-4 | Diagnosis from reports | Text | 1.00 for incidental adrenal nodules, 0.91 for pancreatic cystic lesions, and 0.99 for vascular calcifications. |
| [66] | GPT-4, GPT-3.5 | Report generation | Text | GPT-4=0.997 GPT 3.5= 0.967 |
| [35] | RadBERT, BioBERT, GatorTron | Inference from report | Text | RadBERT, BioBERT, GatorTron-base, and GatorTron-medium LLMs |
|  |  |  |  | achieved F1 scores of 0.84, 0.87, 0.89, and 0.91, respectively |
|  |  |  |  |  |
| **Article** | **Model Used** | **Application of LLM** | **Data Type** | **Other evaluation metrics** |
| [63] | ClinicalBLIP | Report generation | Images | 0.534 |
| [59] | 3D Vision-Language Model | Image analysis and interpretation | Text and 3D images | BLEU: .102, ROUGE-2: .262, BERT: .588 |
| [37] | T5 and Scifive | Inference from report | Text | ROUGE-1:0.816, ROUGE-2:0.668, ROUGE-L: 0.528,, and BLEU: 0.743 |
| [38] | Fine-tuned Text-To-Text Transfer Transformer (T5) model | Report simplification/ summarization | Text | 0.46, 0.28, 0.52, 2.45, and 0.87 in the BLEU-1, METEOR, ROUGE-L, CIDEr, and cosine similarity metrics |
| [42] | PEGASUS, LLaMA and Alpaca | Report generation | Text | 89% of LLM impressions were clinically acceptable by physicians . Mean utility score for LLM impressions was 4.08/5 |
| [54] | GPT-4 | Select imaging modality | Text | agreement: 84% |
| [77] | GPT-4, ChatGPT-3.5, Perplexity, Bing | Report generation | Text | All the models demonstrated good knowledge about |
|  |  |  |  | structured reporting and were able to provide examples of |
|  |  |  |  | structured reports. |
| [46] | Claude.ai, BART, XLM, DeBERTa | Report generation | text | Bart achieved the highest mean similarity score of 99.3%, XLM followed closely with a mean similarity of 98.9% |
| [40] | GPT-4 | Report generation | Text | BLEU Score of 0.5008 |
| [67] | Bloomz-7b1 (a variant of Bloom) | Report simplification/ summarization | Text | BLEU4 ROUGE-L BertScore F1-cheXbert: 25.32 47.48 63.61 74.34 |
| [43] | CXR-IRGen (combining Vision Module and Language Module with LLM) | Report generation | Text and images | BLUE-1(0.3200), BLUE-2 (0.1760), BLUE-3 (0.1066), BLUE-4( 0.0669)  ROUGE-L ( 0.2080) |
| [23] | MMBERT |  | Text and images | BLUE: 69.0 |
| [71] | GPT-3.5 Turbo, GPT-4 | Inference from report | text | Chatbots achieved 86% correct AO codes, lower than radiologists |
| [64] | GPT-3, Show-Attend-Tell | Report generation | Text and images | CIDEr:1.360 , ROUGE_L:0.606, BLEU-1:0.821, BLEU-2:0.672, BLEU-3 BLEU-4: 0.529 0.409 |
| [28] | GPT-4 | Extract information from reports | Text | Concordance 68.8%, , Acceptance 93.8% |
| [69] | GPT-3.5 and GPT-4 | Assess quality | text | Kendall’s Tau (τ): Detailed GPT-4 (5-shot): 0.48 |
|  |  |  |  |  |
|  |  |  |  | Regressed GPT-4 model: 0.64 |
| [58] | GPT-4, Llama 2 | Construct exam questions | Text | Lickert score mean for clarity, relevance, suitability, quality of distractors, and adequacy of rationale for Llama are: 2; 9.9 (±0.4), 9.9 (±0.5), 9.9 (±0.4), 9.8 (±0.5), and 9.9 (±0.3), |
|  |  |  |  | respectively, for GPT-4; and 9.9 (±0.3), 9.9 (±0.2), 9.9 (±0.2), 9.9 (±0.4), and 9.8 (±0.6), |
| [29] | GPT-4 | Image analysis and interpretation | Images | Match rate (62%) |
| [39] | Llama-2-70B-chat | Report generation | Text | Matthews correlation coefficient= 0.75 |
| [75] | GPT-4 | Symptom summarization | Text | median accuracy ratings of 8.45 |
| [47] | GPT-3.5, Bard, Bing, Perplexity | Diagnosis from reports | Images | Perplexity had the highest concordance at 66.67% with expert consensus |
| ([55] | GPT-4 | Answering patients' questions | Text | Responses were rated“appropriate” for 92% of |
|  |  |  |  | 25 tasks and“useful” for 96% |
| [48] | four Text-to-Text Transfer Transformer (T5) | Report simplification/ summarization | Text | ROUGE-1 =57.75 ±30.99, ROUGE-2 = |
|  |  |  |  | 49.96 ±35.36, and ROUGE-L =54.07 ±32.48 in the MIMIC-CXR; and ROUGE-1 =50.00 ±29.24, ROUGE-2 = |
|  |  |  |  | 39.66 ±30.21, and ROUGE-L =47.87 ±29.44 in the JMID. |
| [44] | fine-tuned Bloomz-7b1 | Image analysis and interpretation | Text and images | ROUGE-L =0.373 |
| [20] | Claude 3 Opus, Claude 3.5 Sonnet | Diagnosis from cases | Text and images | The correct diagnosis rates were 58/322 (18.0%) and 69/322 (21.4%), 201/322 (62.4%) and 209/322 (64.9%), and |
|  |  |  |  | 80/322 (24.8%) and 97/322 (30.1%) for Conditions 1, 2, and 3 for Claude 3 Opus and Claude 3.5 Sonnet, respectively. |
| [76] | GPT-4, ChatGPT-3.5 | Report generation | Text | The mean Likert |
|  |  |  |  | score for the AI-LLM–generated radiograph report, CT report, and MRI report was 4.1 ± 0.6, 3.9 ± 0.6, and 3.9 ± 0.7, |
|  |  |  |  | respectively. |
| [65] | GLM-Large, ChatGLM | inference from report | Text | Trained ChatGLM: BLEU-1 BLEU-2 BLEU-3 BLEU-4: 0.7383 0.6477 0.5702 0.5042 |
| [70] | Vicuna-13B | Extract information from reports | Text | κ Median 0.52 to 0.64 |
